# 

**Published:** 1962

**Authors:** 


MEDICAL JOURNAL OF THE SOUTH-WEST

*(Incorporating the Bristol Medico-Chirurgical Journal)*

Established 1883

VOL. 77 (iii–iv) June/October 1962 NOS. 285/286

### Contents

| | | |
|---|---|---|
| CASUALTY SURGEON | *H. K. Bourns* | 69 |
| SURVEY OF A PSYCHOGERIATRIC WARD | *D. M. D. White* | 78 |
| ACUTE ENCEPHALITIS | *Clinico-Pathological Conference* | 81 |
| OBITUARY | | 88 |
| NOTES AND NEWS | | 89 |
| EXAMINATION RESULTS | | 89 |
| APPOINTMENTS | | 90 |
| PROGRAMMES OF MEDICAL SOCIETIES | | 91 |

*PUBLISHED QUARTERLY*

*ANNUAL SUBSCRIPTION* 16/- *POST FREE*

PUBLISHED UNDER THE AUSPICES OF THE
BRISTOL MEDICO-CHIRURGICAL SOCIETY

### *Editorial Committee*

Dr. N. J. Brown, Southmead Hospital, Bristol. *Joint Editor.*

Dr. A. B. Raper, Department of Pathology, Bristol Royal Infirmary. *Joint Editor.*

Dr. J. Macrae, Ham Green Hospital.

Dr. R. C. Wofinden, Central Clinic, Bristol.

Mr. A. E. S. Roberts, Medical Library, University of Bristol. *Secretary.*

### *Local Correspondents*

*Bath* . . Mr. A. D. Bateman, 3 The Circus, Bath.

*Exeter* . . Dr. L. N. Jackson, Mount Jocelyn, Crediton, Devon.

*Gloucester* . Dr. R. F. Jarrett, 9 College Green, Gloucester.

*Plymouth* . Dr. C. R. Croft, Lexden, Hartley Av., Plymouth.

*Taunton* . Dr. M. McAlley, 1 The Crescent, Taunton.

*Torquay* . Dr. A. Robinson Thomas, 20 Devon Square, Newton Abbot, Devon.

*Truro* . . Dr. F. D. M. Hocking, St. Andrews, Perranporth, Cornwall.

*Yeovil* . . Mr. J. A. Vere Nicoll, Knapp House, Yeovil, Somerset.

### NOTICE TO CONTRIBUTORS

Original articles are invited, provided they have not been published elsewhere. Notes of forthcoming events, meetings held, degrees conferred, staff appointments, and other local news are welcomed. The editorial committee reserves the right to make literary corrections.

Illustrations should always be supplied ready for reproduction. All re-touchings or other operations required will be charged to the author. Line drawings should be drawn in Indian ink. We regret that we must ask authors to pay for making blocks, if this is necessary.

J. W. ARROWSMITH LTD., WINTERSTOKE ROAD,
BRISTOL.

### *Notes and News*

BIRTHDAY HONOURS

Macdonald Critchley, C.B.E.
F. E. S. Keiller, M.B.E.

*F.R.C.O.G.:* S. D. Loxton.

Dr. J. A. Fraser Roberts is to give the Leonard Parsons Lectures in 1963 in the University of Birmingham.

Dr. J. H. Middlemiss, appointed first travelling professor of the Faculty of Radiologists R.C.S., visited Africa over a ten-week period beginning in April.

Mr. J. R. Nicholson-Lailey has been elected chairman of council of the B.M.A.

### *Examination Results*

*M.R.C.P.:* S. C. Jordan.

UNIVERSITY OF BRISTOL

*M.D.:* C. Shaldon.
*Ch.M.:* H. J. Espiner.

*D.P.H.*

Amy M. Baird

Valerie N. Baker

D. Cullen

S. Fitil

I. Fracasso

J. O. Koney

J. O. Lloyd-Jones

W. B. Whisker

Graces W. Wu

*M.B., Ch.B.*

SECOND CLASS HONOURS

Patricia A. Clayton
A. J. Drinnan (with distinction in obstetrics and gynaecology)
Linda M. Flint (with distinction in child health)
Wilhelmina M. H. Hughes (with distinction in medicine)

PASS

D. F. Baigent

J. E. Barber

G. R. Burston

V. K. Chaddah

J. R. Coleman

A. M. Dave

Beryl A. Gaythwaite

J. Halazonetis

R. J. Harris

D. F. Kitching

Jennifer I. Lewis

Ninie Limvichitr

D. Lush

S. G. Marsh

B. Mason

M. Messervy

L. F. Moe

J. Nixon

J. O. S. Okeke

J. G. Parmenter

Alison M. L. Pearson

C. A. Pennock

Miriam G. Pope (with distinction in obstetrics and gynaecology)

Pauline A. Primrose

E. M. Ross

G. Sakalis

E. M. Sedgwick

Jennifer M. Shobbrook

M. R. L. Simmons

P. J. Smith

P. J. B. Smith

I. P. Stewart

S. C. H. Tahsin

R. J. Vecht

W. G. Westall

Anne M. Whittingdale

C. B. Williams

Rosemary A. Wood

### Appointments

J. D. Sheward, M.D. (Bristol), consultant paediatrician, mid-Worcestershire and Selly Oak hospital group.

### UNITED BRISTOL HOSPITALS

| Name | Appointment |
|---|---|
| ApSimon, H. T., M.B., CH.B., D.OBST.R.C.O.G. | Senior Registrar in Diagnostic Radiology. |
| Fraser, I. D., M.D. | Senior Registrar in Pathology. |
| Wills, M. R., M.B., CH.B. | Senior Registrar in Chemical Pathology. |
| Bebell, Jean M., M.B., CH.B., D.OBST.R.C.O.G. | Registrar in Obstetrics and Gynaecology. |
| Baskett, P. J. F., M.B., B.CH. | Registrar in Anaesthetics. |
| Wassif, S. B., D.M.R.T. | Registrar in Radiotherapy. |
| Thirkettle, J. L., M.R.C.P. | Registrar in Medicine. |

### SOUTH WESTERN REGIONAL HOSPITAL BOARD BRISTOL

| Name | Appointment |
|---|---|
| Roberts, J. B. M., M.B., CH.B. (Leeds), F.R.C.S. (Eng.). | Senior Surgical Registrar, Department of Urology, Southmead Hospital (Joint appointment with United Bristol Hospitals). |
| Hakes, J. A. A., M.B., CH.B. | Anaesthetic Registrar, Southmead/Frenchay Hospitals. |
| Woodyard, J. E., M.B., CH.B., D.OBST.R.C.O.G., F.R.C.S. (Edin. & Eng.). | Surgical Registrar, Southmead Hospital. |
| Cain, A. A. R., M.A., B.M., B.CH., D.OBST.R.C.O.G., D.C.H. | Medical Registrar, Cossham/Frenchay Hospitals. |
| Cooper, A. J., M.B., CH.B. | Registrar in Psychiatry, Barrow Hospital. |
| Magnus, R. V., M.B., CH.B. | Registrar in Psychiatry, Glenside Hospital. |

### NORTH GLOUCESTERSHIRE

| | |
|---|---|
| Porter, D. S., M.B., B.S. | Surgical Registrar, Cheltenham General Hospital. |
| Thomas, D. H., M.B., B.S. | Surgical Registrar, Gloucestershire Royal Hospital. |

### BATH

| | |
|---|---|
| Veeranna, K., M.B., B.S., D.L.O. (Eng.). | Registrar in E.N.T. Surgery, Bath Group of Hospitals. |

### SOUTH SOMERSET

| | |
|---|---|
| Madden, B. K., M.A., M.B., B.CHIR., F.R.C.S. (Eng.). | Consultant Orthopaedic Surgeon to the South Somerset Clinical Area. |
| Fenn, P. J., M.B., B.S. | Surgical Registrar, Taunton and Somerset Hospital. |
| Rahimtulla, Kulsum A., L.R.C.P.I. & L.M., L.R.C.S.I. & L.M., D.C.H. (Eng.). | Paediatric Registrar, Taunton and Somerset Hospital/The London Hospital (joint appointment with the London Hospital). |

### DEVON AND EXETER

| | |
|---|---|
| Stewart, J. D. M., M.A., M.B., B.CHIR. | Registrar in Orthopaedic and Traumatic Surgery, Torbay Hospital/Princess Elizabeth Orthopaedic Hospital. |
| Jones, A. R., M.R.C.S., L.R.C.P., D.P.M. | Assistant Psychiatrist, Exe Vale Hospital, Exminster, Nr. Exeter. |

### PLYMOUTH

| | |
|---|---|
| Gemmill, Margaret K., M.B., CH.B., D.OBST.R.C.O.G. | Anaesthetic Registrar, South Devon and East Cornwall Hospital, Plymouth. |

*WEST CORNWALL*

| | |
|---|---|
| Anderson, I. F., B.SC., M.R.C.S., L.R.C.P. | Registrar in Obstetrics and Gynaecology, Camborne/Redruth Hospital. |
| Jayaratne, S., M.B., B.S., D.C.H. | Medical Registrar, Royal Cornwall Infirmary, Truro. |

### *Programmes of Medical Societies*

### 1962 CONTENTS OF VOL. 77

| | page |
|---|---|
| Disease of Major Cerebral Vessels. By J. L. G. THOMSON | 1 |
| The Fascial Flap Operation for Inguinal Hernia. By EDRIC WILSON | 7 |
| Open Cardiac Surgery during Profound Hypothermia. By A. J. G. DICKENS | 11 |
| Tranquillizers. By R. E. HEMPHILL | 18 |
| Malignant Ependymoma masquerading as Tuberculous Meningitis (Clinical Pathological Conference) | 28 |
| Iatrogenic Liver Disease. By A. E. READ | 41 |
| Congenital Malformations: Clinical Considerations. By BERYL D. CORNER | 46 |
| Congenital Malformations: Aetiology and Pathology. By N. J. BROWN | 53 |
| Unusual Type of Bacterial Endocarditis in a case of Myocardial Infarction (Clinical Pathological Conference) | 60 |
| Casualty Surgeon. By H. K. BOURNS | 69 |
| Survey of a Psychogeriatric Ward. By D. M. D. WHITE | 78 |
| Acute Encephalitis (Clinical Pathological Conference) | 81 |
| OBITUARY: | |
| J. W. E. Snawdon | 35 |
| H. H. Carleton | 36 |
| G. Macleod | 88 |
| NOTES AND NEWS | 38, 65, 89 |
| PROGRAMMES OF MEDICAL SOCIETIES | 91 |
| EXAMINATION RESULTS | 89 |
| APPOINTMENTS | 38, 65, 90 |
| NEWS FROM THE SOCIETIES | 39, 67 |

INDEX MEDICUS

NATIONAL LIBRARY

MAR 21 1963

MEDICINE

### 1962 INDEX TO VOL. 77

Bacterial Endocarditis in a case of Myocardial Infarction—Clinical Pathological Conference 60
Bourns H. K.—Casualty Surgeon 69
Brown N. J.—Congenital Malformations, Aetiology & Pathology 53

Casualty Surgeon—H. K. Bourns, 69
Cerebral Vessels, major, disease of—J. L. G. Thomson, 1
Clinical Pathological Conferences:
—Acute Encephalitis, 81
—Malignant Ependymoma masquerading as Tuberculous Meningitis, 28
—Unusual type of Bacterial Endocarditis in a case of Myocardial Infarction, 60
Congenital Malformations, Aetiology and Pathology—N. J. Brown, 53
Congenital Malformations, Clinical Considerations—Beryl D. Corner, 46
Corner, Beryl D.—Congenital Malformations, Clinical Considerations, 46
Dickens, A. J. G.—Open Cardiac Surgery during Profound Hypothermia, 11

Encephalitis, Acute—Clinical Pathological Conference, 81

Ependymoma, malignant masquerading as Tuberculous Meningitis—Clinical Pathological Conference, 28

Fascial Flap operation for Inguinal Hernia E. Wilson, 7

Hemphill, R. E.—Tranquillizers, 18

Iatrogenic Liver disease—A. E. Read, 41

Open Cardiac Surgery during profound Hypothermia—A. J. G. Dickens, 11

Psychogeriatric Ward, Survey of—D. M. D. White, 78

Read, A. E.—Iatrogenic Liver Disease, 41

Thomson, J. L. G.—Disease of Major Cerebral Vessels, 1
Tranquillizers—R. E. Hemphill, 18

White, D. M. D.—Survey of a Psychogeriatric Ward, 78
Wilson, E.—Fascial Flap operation for Inguinal Hernia, 7

